# L-3-n-Butylphthalide Protects HSPB8 K141N Mutation-Induced Oxidative Stress by Modulating the Mitochondrial Apoptotic and Nrf2 Pathways

**DOI:** 10.3389/fnins.2017.00402

**Published:** 2017-07-12

**Authors:** Xiao-Dong Yang, Zhi-Dong Cen, Hai-Peng Cheng, Kai Shi, Jie Bai, Fei Xie, Hong-Wei Wu, Bei-Bei Li, Wei Luo

**Affiliations:** ^1^Department of Neurology, The Second Affiliated Hospital, School of Medicine, Zhejiang University Hangzhou, China; ^2^Department of Neurology, Ruijin Hospital, School of Medicine, Shanghai Jiao Tong University Shanghai, China; ^3^Department of Neurology, Northwestern University Feinberg School of Medicine Chicago, IL, United States; ^4^Department of Neurology, Qingdao Municipal Hospital, School of Medicine, Qingdao University Qingdao, China; ^5^Department of Neurology, Qingdao No.8 People's Hospital Qingdao, China

**Keywords:** small heat shock protein HSPB8, Charcot-Marie-Tooth disease, mitochondrial dysfunction, oxidative stress, L-3-n-butylphthalide

## Abstract

Charcot–Marie–Tooth disease (CMT), also known as hereditary motor and sensory neuropathy, is the most common inherited peripheral nerve disorder. Missense mutations, such as K141N, in the small heat shock protein HSPB8 are known to cause distal hereditary motor neuropathy 2A (dHMN2A) or Charcot-Marie-Tooth neuropathy type 2L (CMT2L). However, of critical clinical significance, very few specific therapies for this disease exist. In the present study, we investigated the impact of mutant K141N HSPB8 on mitochondrial distribution and function in a cellular model of CMT2L. Our results indicate that K141N HSPB8 induced mitochondrial aggregation and caused increased oxidative stress injury. As an extraction from Chinese celery Apium graveolens Linn seeds, L-3-n-Butylphthalide (NBP), has been reported to exert many neuroprotective effects, we interrogated whether NBP could elicit a protective effect on the cell injury typically caused by HSPB8 K141N mutations. We found NBP could reverse the pathological processes induced by HSPB8 K141N mutation via an antioxidant effect, modulation of the Bax/Bcl-2 mitochondrial apoptotic and Nrf2 pathways. We propose a novel function of HSPB8, highlighting the consequence of the K141N pathogenic mutation. Furthermore, we suggest NBP may have promising therapeutic potential in the treatment of CMT2L.

## Introduction

Charcot–Marie–Tooth disease (CMT), also known as hereditary motor and sensory neuropathy, is the most common inherited peripheral nerve disorders. The disease usually becomes apparent in adolescence or early adulthood and is characterized by progressive muscle weakness and atrophy, sensory loss, foot (and hand) deformities and steppage gait (Szigeti and Lupski, 2009). CMT is conventionally divided into a demyelinating form (CMT1) and an axonal defective form (CMT2). CMT1 exhibits markedly reduced nerve conduction velocities (NCVs), whereas CMT2 shows slightly reduced or normal NCVs (Harding and Thomas, 1980). Currently, there exists limited therapeutic recourse for those affected with this disease.

Missense mutations (K141N, K141E, and K141T) in the α-crystallin domain of the small heat shock protein HSPB8 (HSP22) cause distal hereditary motor neuropathy 2A (dHMN2A) or Charcot-Marie-Tooth neuropathy type 2L (CMT2L) (Irobi et al., 2004; Tang et al., 2005; Nakhro et al., 2013). HSPB8 is a stress-responsive member of the superfamily of small heat shock proteins, which has multiple cellular functions including: chaperone activity, anti-oxidation, apoptosis, anti-apoptosis and lifespan extension (Kappé et al., 2003; Shemetov et al., 2008).

Large amounts of energy are required for neurons to carry out their highly specialized functions (Chen and Chan, 2006). Thus, mitochondria as sources of cellular energy, play an integral role in neuronal functions. Targeting mitochondria protection pathways have been found to be a potential therapeutic approach for ischemia and neurodegenerative diseases (Dirnagl and Meisel, 2008; Camilleri and Vassallo, 2014; Lukyanova and Kirova, 2015). However, the specific role of the HSPB8 K141N mutation on mitochondria dysfunction has yet to be investigated. Previous studies have found that cell injury, neurite degeneration and protein degradation occur as a consequence of a HSPB8 mutation (Irobi et al., 2004, 2010; Kwok et al., 2011). Therefore, drugs that can prevent cell apoptosis, inhibit neurite degeneration or mitochondrial dysfunction may be an appropriate choice for the treatment of CMT2L. As there is no potential neuroprotective treatments CMT2L, a more thorough understanding of the mechanisms of pathogenesis could help in the development of targeted therapies.

L-3-n-butylphthalide (NBP) is extracted as a pure component from seeds of Apium and was approved by the State Food and Drug Administration (SFDA) of China for clinical use in patients with stroke in 2002. The beneficial effects of NBP have been well-established in a variety of *in vivo* and *in vitro* studies modeling stroke (Liu et al., 2007; Xu J. et al., 2012), Alzheimer's disease, and Parkinson's disease (Huang et al., 2010; Xiong et al., 2012). NBP exerts a protective effect on these diseases via multiple mechanisms including, but not limited to: improving energy metabolism (Feng et al., 1995), enhancing angiogenesis and improving cerebral microvessels growth (Liao et al., 2009), protecting endothelial cells against mitochondrial damage and subsequent cell death (Li et al., 2009), reducing neuronal apoptosis and maintaining structure and morphology of neurons (Ma et al., 2009). While the protective effect of NBP highlights its role as a potential candidate for the treatment of CMT2L, no studies to date have specifically addressed this hypothesis. As such, the aims of the present study were to explore the relationship between mutant HSPB8 and mitochondria, in addition to investigating the protective effect of NBP against HSPB8 K141N mutation caused neurotoxicity in cultured neuroblastoma SH-SY5Y cells.

## Materials and methods

### Materials and treatment

Neurobasal medium, B27 supplement, nerve growth factor (NGF), trypsin, horse serum, Dulbecco's modified Eagle's medium (DMEM) and fetal bovine serum (FBS) were purchased from Gibco BRL (New York, NY, USA). Anti-HSPB8 primary antibody was purchased from Abcam (Cambridge, CB, UK). Anti-β-actin, anti-Bcl-2, anti-Bax, anti-Nrf2 and anti-Lamin B primary antibodies and anti-rabbit/mouse IgG secondary antibodies were purchased from Santa Cruz Biotechnology (Santa Cruz, CA, USA). 2,7-Dichlorofluorescin-diacetate (DCFH-DA), and poly-D-lysine were purchased from Sigma–Aldrich (St. Louis, MO, USA). Mito-Tracker Red was purchased from Invitrogen (Carlsbad, CA, USA). NBP (generous gift from Shijiazhuang Pharmaceutical Group Ouyi Pharma Co., Ltd.) was dissolved in DMSO before dilution with the culture medium. The final concentration of DMSO per well was 0.2%. Cells were pre-incubated with NBP for 24 h prior to transduction. After transfection we added NBP into the medium until for further studies.

### Primary cell culture and quantification of neurites

Animal procedures were approved by the Animal Experimentation Ethics Committee of Zhejiang University. Spinal motor neuron cultures were prepared according to previously described protocols (Van Damme et al., 2007; Irobi et al., 2010) with minor modifications. Spinal cords were dissected from embryonic day 13–14 Sprague-Dawley rats. The meninges and dorsal root ganglia were removed, and the ventral spinal cords were cut into pieces and dissociated using trypsin/EDTA. Following centrifugating on a 6.2% iodixanol OptiPrep cushion, motor neurons were separated from the glial cells and small neurons, and seeded on coverslips coated with poly-D-lysine and laminin at a density of 7,000–10,000 cells/cm^2^. Neurons were cultured in Neurobasal plating medium supplemented with 2% B-27, 4 g/L glucose, 10% horse serum, 2 mmol/L L-glutamine. All plating medium was replaced 1 h after plating with Neurobasal maintenance medium supplemented with 2% B-27, 4 g/L glucose, 2 mmol/L L-glutamine, 2% horse serum, and 20 ng/ml NGF. After 2 days in culture, 2.5 μmol/L AraC was added for 48 h to inhibit the outgrowth of non-neuronal cells. Cells were then shifted into a maintenance medium identical to the plating medium but lacking fetal serum. Half of the culture medium was replaced every 3 days. On day 3, transduction were performed using pLVX-Puro vectors, and the culture medium was changed 24 h post-transduction. GFP reporter via fluorescent microscopy was used to monitor transduction efficiency, which was greater than 60%. As described previously (Irobi et al., 2010), neurofilament heavy antibody SMI32 (Sternberger monoclonals dilution 1/1,000) and the specialized morphology of motor neurons [neuronal cells with large pyramidal soma (diameter > 25 μm) which have more than two neurites] were used to discriminate motor neurons from other cell types during neurite quantification. Immunofluorescence images were collected on a FluoView FV1000 confocal microscope (Olympus, Tokyo, Japan). Ninety (*n* = 90) motor neurons from three independent experiments in randomized fields were analyzed in details for neurite number. Cell counts were done blinded and all experiments were repeated independently.

### SH-SY5Y cell culture and transfection

SH-SY5Y cells were maintained in DMEM with 10% FBS. Cells were transfected with either an empty vector, or a vector with wild-type (WT) or K141N mutant human HSPB8 cDNA using Lipofectamine™2000 transfection reagent (Invitrogen Carlsbad, CA, USA), according to the manufacturer's protocol. All cell lines were routinely maintained in a 5% CO_2_ incubator at 37°C, changing the medium every 2–3 days. Cultured cells were used for treatment 48 h post-transfection.

### Immunocytochemistry

Transfected cells were stained in the dark with Mito-Tracker Red (100 nmol/L) for 30 min, and washed twice for 2 min with PBS. Cells were fixed with 4% paraformaldehyde for 15 min at room temperature. The coverslips were washed with PBS, permeabilized for 15 min in 0.2% TritonX-100, and washed again. The coverslips were subsequently incubated with DAPI for 15 min, washed with PBS, adhered to microscope slides and stored in the dark at 4°C until use. Immunofluorescence images were collected on a FluoView FV1000 confocal microscope (Olympus, Tokyo, Japan). The proportion of cells with protein aggregates and mitochondrial aggregates were calculated 48 h after transfection. Approximately 300 independent SH-SY5Y cells from three independent experiments in randomized fields were analyzed in details for mitochondrial aggregation. To quantify the ratio of HSP8 colocalization with mitochondria, an assessment of GFP and MitoTracker colocalization within a single cell and comparing ROI (region of interest) containing GFP and Mito Tracker with ROI only containing Mito Tracker was made using ROI color analysis with specific software (Fiji Is Just ImageJ, Tokyo, Japan).

### Western blot

Cells were collected and rinsed twice with PBS and lysed with cells lysis buffer. The isolation of cytosolic and nuclear proteins was performed using nuclear and cytoplasmic protein extraction kit purchased from Applygen Technologies Inc. (Beijing, China). Protein extracts were quantified using a BCA kit purchased from Beyotime Institute of Biotechnology (Shanghai, China). Equal quantities of protein samples were loaded onto SDS-PAGE gels and transferred onto PVDF membranes (Millipore). After blocking with 5% non-fat milk, the membranes were incubated overnight at 4°C with primary antibodies in TBST. The following day, after extensive washing in TBST, the membranes were incubated with secondary antibodies. Immunoreactive bands of interest were scanned using an Odyssey Infrared Imaging System (LI-COR, Lincoln, NE, USA).

### Measurement of intracellular reactive oxygen species (ROS)

DCFH-DA was used to estimate ROS. Briefly, cells were incubated with DCFH-DA for 20 min in the dark at 37°C. Following treatment, cells were washed with PBS twice and immediately examined by flow cytometry to determine fluorescence intensity with excitation wavelength at 505 nm and emission wavelength at 534 nm. Non-transfected cells were used as the normal control. ROS were expressed as a percentage of the control. In each analysis at least 10,000 cells were recorded.

### Measurement of the content of H_2_O_2_

The content of H_2_O_2_ was assessed using a commercially available kit (Nanjing Jiancheng Bioengineering Institute). H_2_O_2_ bound with molybdenic acid to form a complex, which was measured at 405 nm and the content of H_2_O_2_ was then calculate.

### Measurement of superoxide dismutase (SOD) and catalase (CAT) activities

SOD activity was studied using water-soluble tetrazolium salt (WST-1) method with the commercially available kit (Dojindo Laboratories, Kumamoto, Japan) according to the manufacture's protocol. Briefly, after treatment, the cell lysate was centrifuged and then protein level was quantified. Cell lysates (50 μg protein/ml) were subjected for assay. CAT activity was measured using catalase activity assay kit (Beyotime Biotechnology) as per the manufacturers instructions. The developed red color product, N-4-antipyryl-3-chloro-5-sulfonate-p-benzoquinonemonoimine, was absorbed maximally at 520 nm.

### Measurement of the mitochondrial transmembrane potential

Mitochondrial membrane potential (MMP) were assessed using JC-1 dye (Beyotime Biotechnology). Cells were incubated with the medium containing JC-1 (5 μg/ml) for 30 min. After washing, MMP were monitored by determining the relative amounts of dual emissions from mitochondrial JC-1 monomers or aggregates using a flow cytometer under 488 nm laser excitation. The green fluorescence represents JC-1 monomers when MMP is low, whereas red fluorescence represents JC-1 aggregates when MMP is high. We used the red/green fluorescence ratio to represent MMP.

### Cell viability detection by CCK-8

SH-SY5Y cells were seeded in a 96-well-plate at 5000 cells/well. After treatment with NBP and transfection, 10 μL of CCK-8 (Obio Technology, Shanghai) was added to the culture medium. The viability of the cells was measured 4 h later at 450 nm using a Microplate reader (Synergy Mx, Bio-Tek, America) according to the manufacturer's instructions.

### Transmission electron microscopy (TEM)

Sural nerves were obtained from a CMT2L patient who was diagnosed carrying HSPB8 K141N mutation and one age matched control who was diagnosed with diabetes. The participants signed the informed consent, and the Institutional Review Board from the Zhejiang University approved the study. For transmission electron microscopy, the specimens were fixed with 2.5% glutaraldehyde, washed with PBS, rinsed in 0.1 M phosphate buffer for 30 min and fixed in 1% osmic acid for 1 h at 4°C. The specimen was then, rinsed again with the phosphate buffer for 30 min, treated with 2% uranyl acetate for 30 min, and subjected to progressive dehydration prior to embedding in Epon 812 resin. The embedded specimens were sectioned in parallel at 90 nm with an ultramicrotome (UCT, Leica, Germany), stained sequentially with uranyl acetate and lead citrated and viewed under TEM (TECNAI-10; Philips, Amsterdam, Netherlands) using a 50-mm aperture, 80 kV acceleration.

### Statistical analysis

The data were analyzed using one-way ANOVA (analysis of variance) followed by Tukey's *post hoc* test for multiple comparisons where necessary. Values shown represent mean ± SEM of at least three independent experiments. Differences were considered significant when *P* < 0.05. The analyses were performed using SPSS 16.0 software.

## Results

### HSPB8 K141N mutation caused mitochondrial aggregation

**Figure 1:**
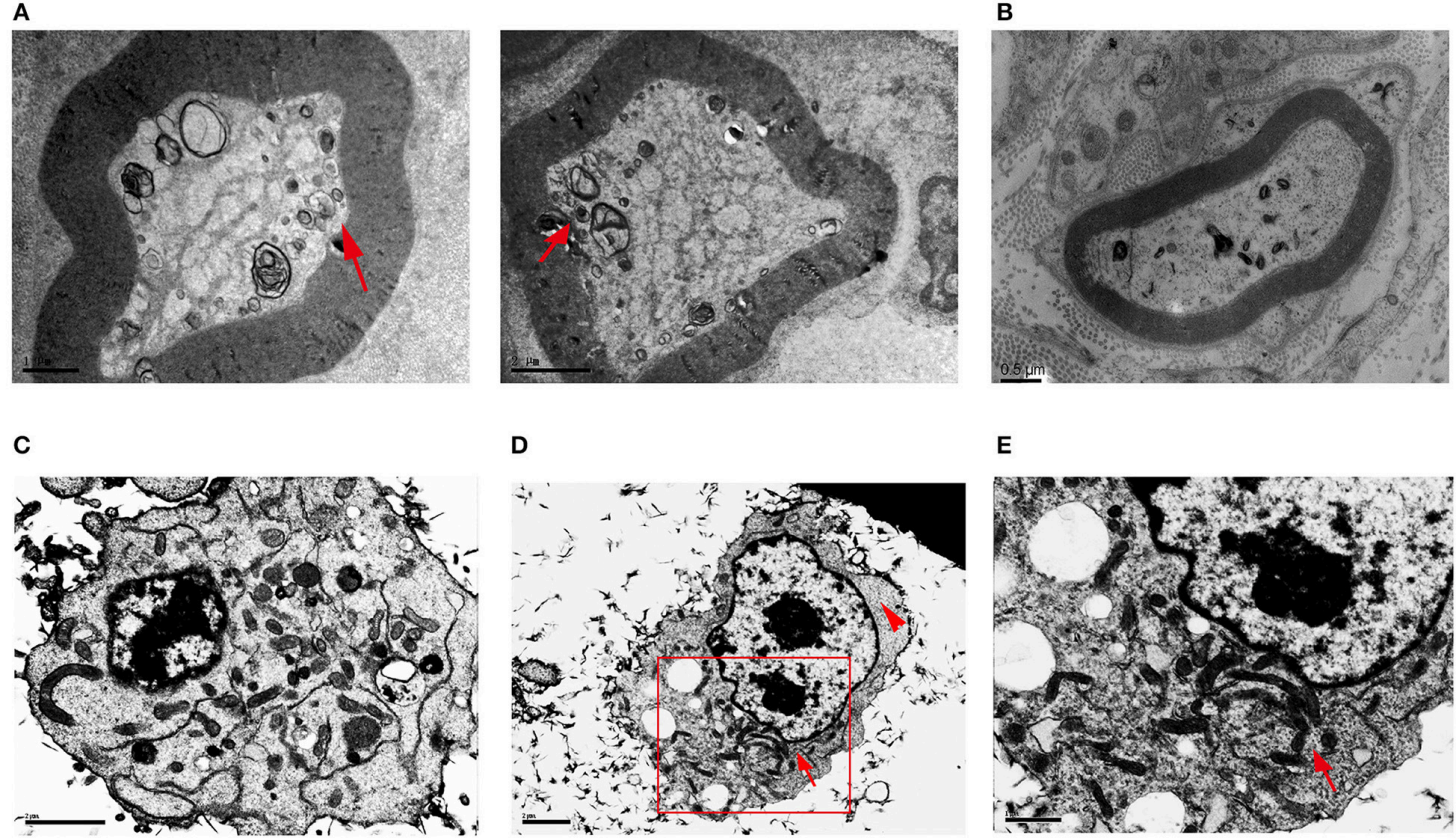
Transmission electron microscopy of sural nerves and HSPB8 transfected SH-SY5Y cells. **(A)** Sural nerves from a CMT2L patient. Arrow shows the abnormal mitochondrial membranes, aggregated, small, round mitochondria. **(B)** Sural nerves from diabet patient. **(C)** SH-SY5Y cells expressing WT HSPB8 have normal mitochondrial distribution with a discrete appearance that was diffuse throughout the cell. **(D)** SH-SY5Y cells expressing K141N HSPB8 show aggregated mitochondria in either one or two large clusters, as shown by the arrow. The arrowhead shows cellular regions devoid of mitochondria. **(E)** Higher magnification of aggregated mitochondria of **(D)** shows apparently aggregates of individual mitochondria. Scale bars **(A,B)**: 1, 2, and 0.5 μm respectively, **(C–E)**: 2, 2, and 1 μm respectively.

To explore whether HSPB8 K141N mutation influence mitochondria localization and appearance, we studied sural nerves from CMT2L patient by electron microscopy. In the patient's sural nerve fibers there were aggregated mitochondria in the axon while prominent mitochondrial aggregates were found at the periphery of the axon. The size of mitochondria was smaller than normal, while the shapes were round instead of the expected tubular shape (arrows, Figure 1A). While in sural nerves of human diabetic patient, we could not find mitochondrial aggregation (Figure 1B).

To further verify our results, we used neuroblastoma SH-SY5Y cells transfected with WT and K141N HSPB8. The TEM results demonstrated that cells overexpressing WT HSPB8 exhibited ubiquitous mitochondrial distribution throughout the cell (Figure 1C), while K141N-overexpressing cells induced mitochondria aggregation into either one or two large clusters (arrows, Figures 1D,E) and resulted in some cellular regions devoid of mitochondria (arrow heads, Figure 1D).

**Figure 2:**
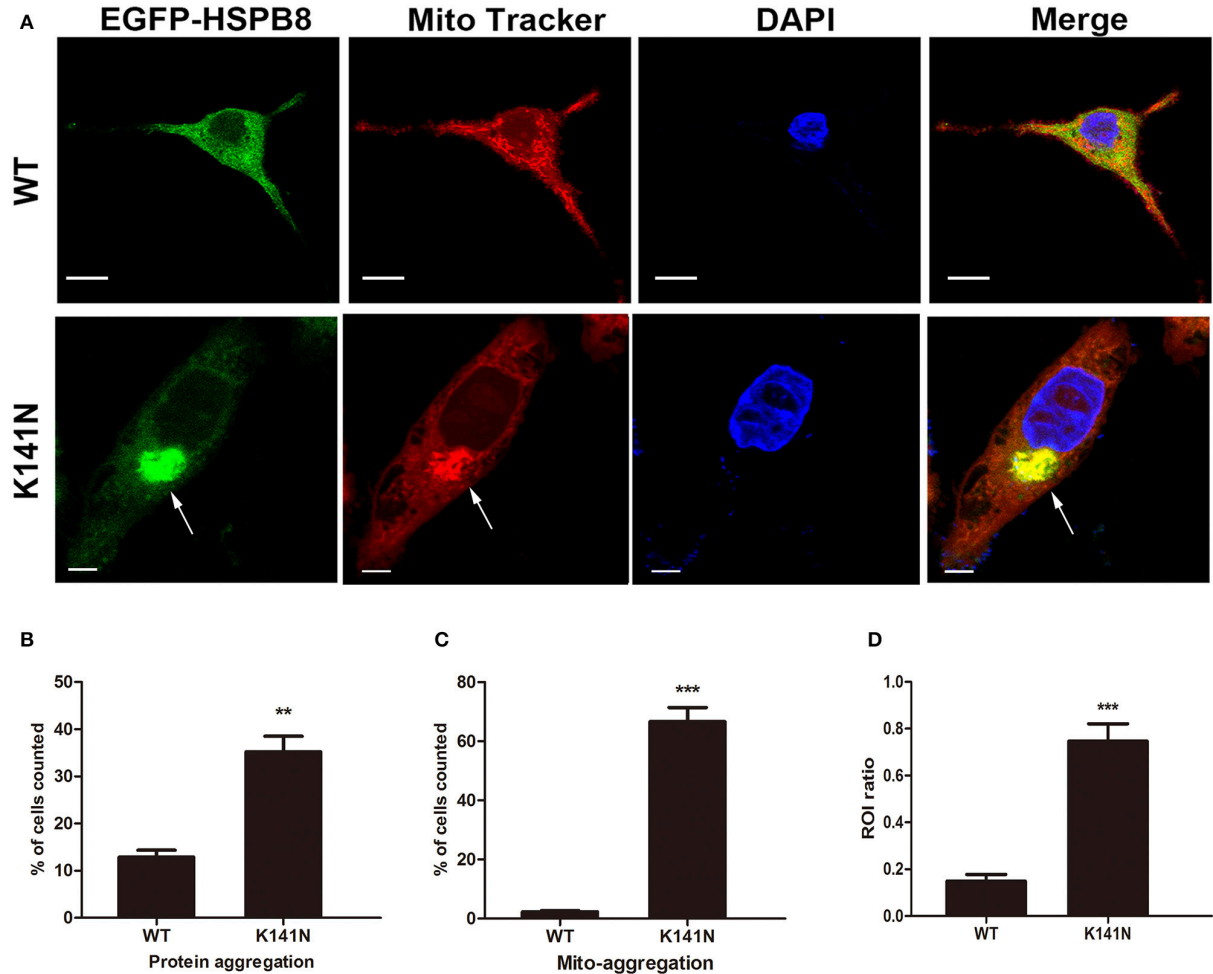
K141N HSPB8 induced mitochondria aggregation in SH-SY5Y cells. **(A)** Representative confocal microscopic images of SH-SY5Y cells transfected with WT or K141N HSPB8 constructs. Nucleus is shown in blue, arrows show the aggregated mitochondria (red) and its co-localization with aggregated K141N HSPB8 (green). Scale bars = 10 μm. **(B,C)** Quantitative analysis of the ratio of K141N HSPB8 proteins to form aggregates and the aggregated mitochondrial co-localization with HSPB8. **(D)** Quantitative analysis of the ROI ratio in WT and K141N HSPB8 groups. Error bars represent ± SEM values and *n* = 3. Approximately 100 transfected cells were counted in each experiment. ^**^p < 0.01, ^***^p < 0.001 compare to the WT HSPB8.

To further explore the relationship between the K141N mutation and mitochondria, we introduced WT HSPB8 or K141N mutant HSPB8 into SH-SY5Y cells using recombinant plasmids in which GFP was fused to the N-terminus of HSPB8. Mitochondria were visualized using Mito-Tracker Red staining. Consistent with a previous studies (Fontaine et al., 2006), expression of K141N increased HSPB8 protein aggregates as compared to WT HSPB8. We also observed varying degrees of mitochondrial aggregation in SH-SY5Y cells expressing K141N HSPB8. Interestingly, overexpression of K141N co-localized with aggregated mitochondria, while WT HSPB8 induced less mitochondrial aggregation in SH-SY5Y cells (Figure 2A). To quantify the difference of protein aggregates and the aggregated mitochondria, we counted the cells containing HSPB8 protein aggregates and the mitochondrial aggregates co-localized with HSPB8, and calculated the proportion of positive cells. As shown in Figure 2B, K141N overexpression significantly increased the percentage of cells with aggregated HSPB8 proteins (*p* < 0.01) and the aggregated mitochondria (*p* < 0.001, Figure 2C) as compared to wild-type controls. To quantify the ratio of HSPB8 colocalization with mitochondria, an assessment of GFP and MitoTracker colocalization within a single cell and comparing ROI containing GFP and Mito Tracker with ROI only containing Mito Tracker was made. Comparing with WT HSPB8, more mitochondria was colocalized with K141N HSPB8 (*p* < 0.001, Figure 2D). These data suggests that K141N HSPB8 may increase mitochondrial aggregation.

### HSPB8 K141N mutation induced mitochondrial dysfunction and increased oxidative stress injury

**Figure 3:**
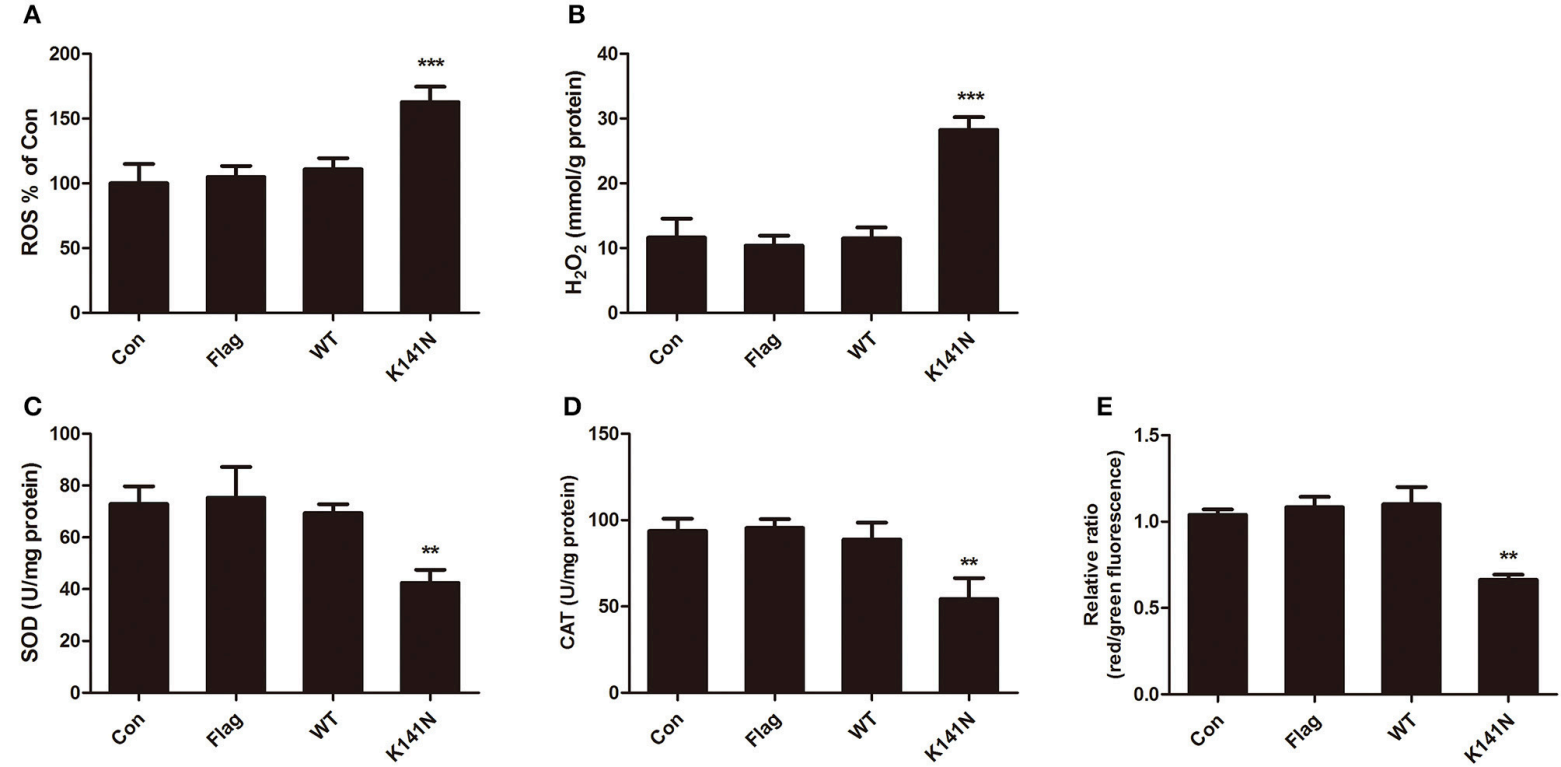
HSPB8 K141N mutation increased mitochondria oxidative stress injury. ROS **(A)** and H_2_O_2_ **(B)** were measured in SH-SY5Y cells transfected with WT HSPB8 and K141N HSPB8. HSPB8 K141N mutation increased intracellular ROS formation (*n* = 3, *p* < 0.001) and H_2_O_2_ content (*n* = 3, *p* < 0.001). SOD **(C)** and CAT **(D)** activities were measured in SH-SY5Y cells transfected with WT HSPB8 and K141N HSPB8. HSPB8 K141N mutation decreased the activities of SOD (*n* = 3, *p* < 0.001) and CAT (*n* = 3, *p* < 0.01). **(E)** MMP was assayed by JC-1 staining. HSPB8 K141N mutation decreased the red/green fluorescence ratio (*n* = 3, *p* < 0.01). SOD: Superoxide dismutase; CAT: Catalase; MMP: Mitochondrial membrane potential. Error bars represent ± SEM values. ^**^p < 0.01, ^***^p < 0.001 compared to the WT HSPB8.

To investigate whether the mitochondrial functions were affected by aggregation in K141N-expressing SH-SY5Y cells, we measured the contents of ROS and H_2_O_2_ and the activities of antioxidant enzymes superoxide dismutase (SOD) and catalase (CAT) in SH-SY5Y cells, these are key markers of oxidative stress. There was no significant difference between the WT HSPB8 group and controls. While compared with the WT HSPB8 group, there was a significant increase in ROS level (*p* < 0.001, Figure 3A) and H_2_O_2_ content (*p* < 0.001, Figure 3B) and a decrease in SOD (*p* < 0.001, Figure 3C) and CAT (*p* < 0.01, Figure 3D) activities in the K141N HSPB8 group. We then measured mitochondrial membrane potential (MMP) to assess the effect of HSPB8 K141N mutation on energy related mitochondria dysfunction. As shown in Figure 3E, the red to green fluorescence ratio decreased in K141N HSPB8 overexpression group as compared to wild-type controls, indicating a decrease in MMP.

### NBP inhibited HSPB8 K141N mutation induced neurotoxicity by modulating the mitochondrial apoptosis pathway

**Figure 4:**
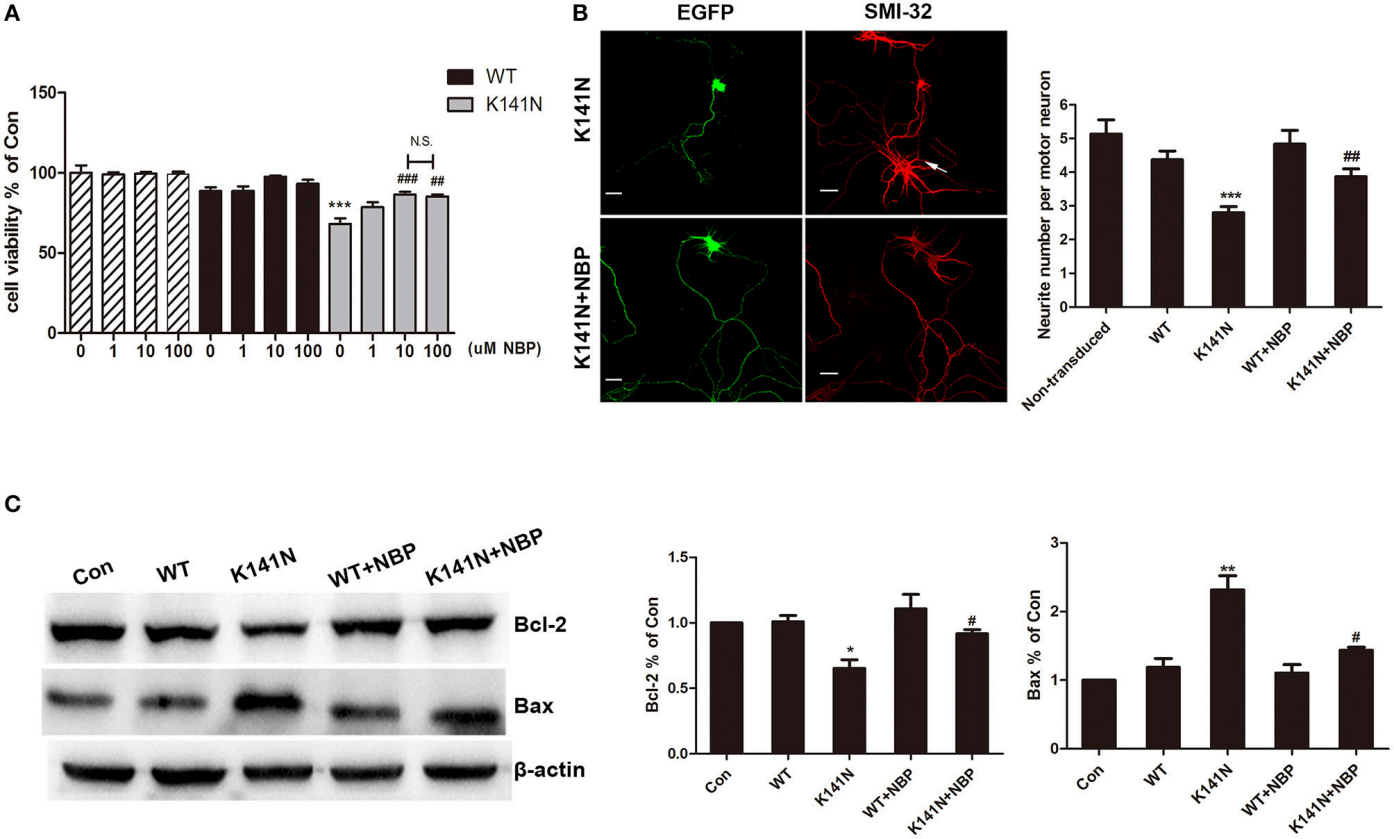
NBP inhibited HSPB8 K141N mutation induced neurotoxicity by modulating the mitochondrial apoptosis pathway. **(A)** The effect of NBP with different concentration on cell viability was shown (*n* = 6). **(B)** Representative confocal microscopic images of spinal motor neurons transduced with HSPB8. Counts of the number of neuritis was performed (*n* = 3, about 30 transduced cells were counted in each experiment). HSBP8 was shown in green, and the arrow showed the non-transduced neurons, scale bars = 10 μm. **(C)** Representative Western blot experiments of Bcl-2 and Bax protein in NBP pre-treatment and HSPB8 transfected SH-SY5Y cells. A decreased in Bcl-2 protein level and an increased in Bax protein level were observed in the HSBP8 K141N over-expression group. NBP treatment decreased the ratio of Bax/Bcl-2. Protein levels were quantified by densitometric analysis and normalized to β-actin. Values are means of at least three different blots. The error bars indicate ± SEM, ^*^p < 0.05, ^**^p <0.01 and ^***^p < 0.001 compared to the WT HSPB8. ^#^*p* < 0.05, ^##^*p* < 0.01 and ^###^*p* < 0.001 compared to K141N group.

The protective effect of NBP against K141N mutation-induced cytotoxicity was assessed using the CCK8 method. K141N mutants significantly reduced cell viability, as compared with wild-type HSPB8, measured 48 h following transfection. NBP had no detectable toxic effects and did not affect the cell viability of WT HSPB8 transfected cells (Figure 4A). We did observed an attenuation of cell damage in a concentration-dependent manner in K141N mutants pre-incubated with 10, and 100 μmol/L NBP. Notably, effect of NBP at 100 μmol/L was not significantly different from that observed at 10 μmol/L, suggesting that 10 μmol/L NBP is the maximal protective dose. As such, we chose 10 μmol/L NBP as the dose used for the subsequent tests (Figure 4A).

NBP can also inhibit HSPB8 K141N mutation induced neurotoxicity in primary spinal motor neurons. Consistent with previous reports (Irobi et al., 2010), overexpression of GFP-tagged HSPB8 K141N protein led to a reduction in the number of neurites in spinal motor neurons as compared to non-transduced neurons (arrow show) (*p* < 0.001, Figure 4B). Following a treatment of NBP the number of neurite increased and a significant difference pre- and post-treatment was observed (*p* < 0.01, Figure 4B).

Bcl-2 family is a principal regulator of the mitochondrial apoptotic program, and thus we used western blot to examine the expressions of Bcl-2 and pro-apoptotic protein Bax (Figure 4C). Overexpression of HSPB8 K41N protein decreased the expression level of Bcl-2 (*p* < 0.05) while a concomitant increase in the level of Bax was observed (*p* < 0.01) as compared with overexpression of wild-type HSPB8. However, treatment of NBP at a concentration of 10 μmol/L robustly increased Bcl-2 levels (*p* < 0.05) and decreased Bax levels (*p* < 0.05). Taken together, these results suggest that treatment of NBP could significantly attenuate HSPB8 K141N-induced neurotoxicity by up-regulating the ratio of Bax/Bcl-2.

### NBP inhibited K141N-induced oxidative stress through activation of the Nrf2 pathway in SH-SY5Y cells

**Figure 5:**
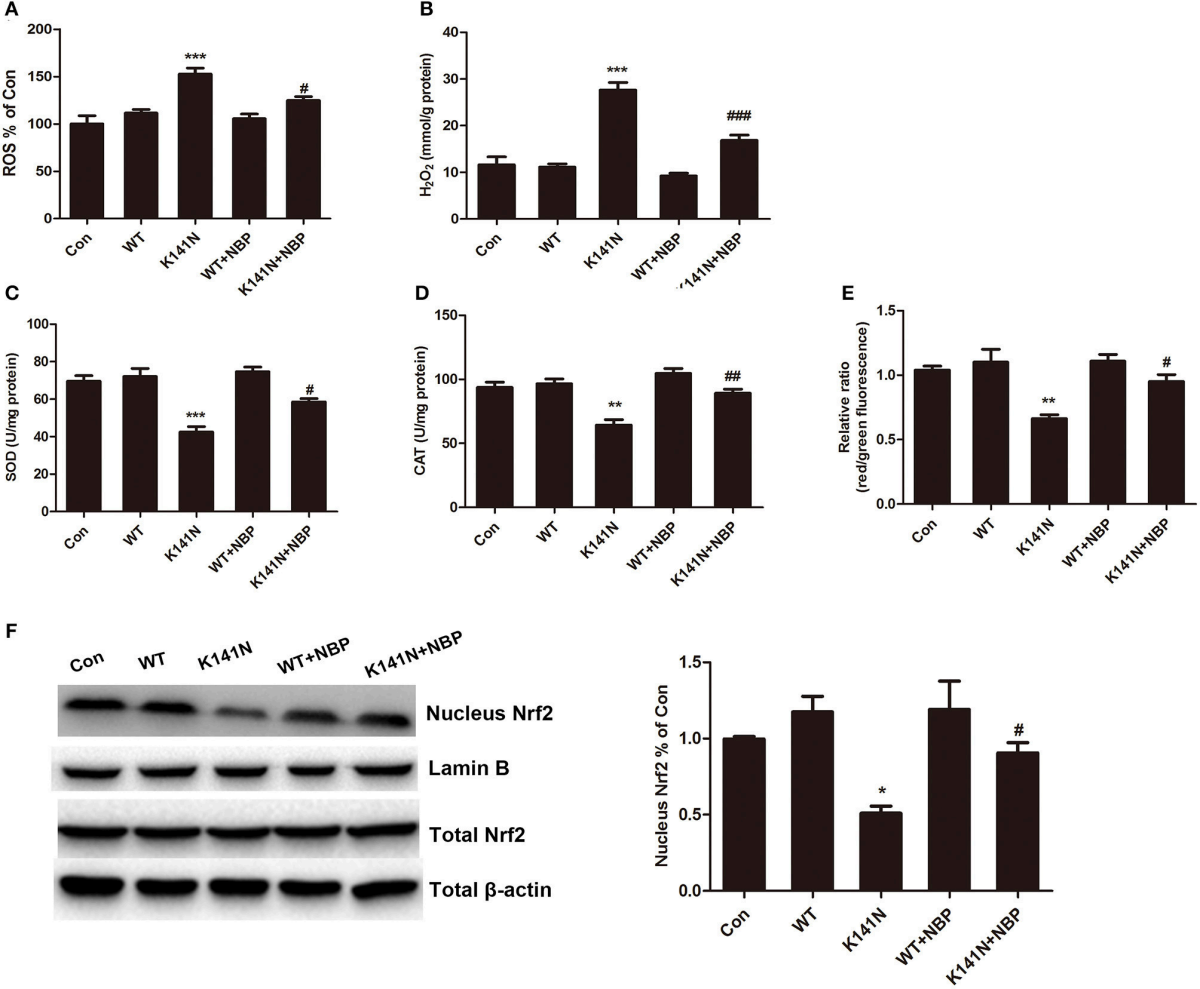
NBP had a protective effect on K141N HSPB8 induced mitochondrial dysfunction through the Nrf2 pathway. Treatment with 10 μmol/L NBP reduced the **(A)** ROS levels (*n* = 3, *p* < 0.05) and **(B)** H_2_O_2_ content (*n* = 3, *p* < 0.001) compared with K141N groups. Treatment with 10 μmol/L NBP increased the activity of **(C)** SOD (*n* = 3, *p* < 0.05) and **(D)** CAT (*n* = 3, *p* < 0.01) compared with K141N groups. **(E)** Treatment with 10 μmol/L NBP prevented the K141N-induced MMP reduction (*n* = 3, *p* < 0.05). **(F)** Total cellular protein and nuclear protein fractions were extracted and expression levels of Nrf2 were detected by Western blotting. β-actin was used as a loading control for total protein fraction, while Lamin B was used as a loading control for the nuclear protein fractions. Values are means of at least three different blots. SOD: Superoxide dismutase; CAT: Catalase; MMP: Mitochondrial membrane potential. Error bars represent ± SEM values. ^*^p < 0.05, ^**^p < 0.01, ^***^p < 0.001 vs. the WT HSPB8. ^#^*p* < 0.05, ^##^*p* < 0.01 and ^###^*p* < 0.001 vs. K141N group.

We next investigated whether NBP had a protective effect on HSPB8 K141N mutation-induced mitochondrial dysfunction in SH-SY5Y cells. The results showed that NBP could inhibit K141N-induced oxidative damage. As shown in Figure 5A, 10 μmol/L NBP treatment significantly decreased the level of intracellular ROS (*p* < 0.05) and H_2_O_2_ content (*p* < 0.001, Figure 5B), increased the activities of SOD (*p* < 0.05, Figure 5C) and CAT (*p* < 0.01, Figure 5D), and prevented the K141N-induced MMP reduction in SH-SY5Y cells (*p* < 0.01, Figure 5E).

Treatment with NBP inhibited K141N-induced oxidative stress suggesting that it might enhance intracellular antioxidant defense. We further to explore the underlying mechanism. It is well-established that Nrf2 is a transcription factor that induces antioxidant gene expression by binding to the antioxidant response element (ARE) located in the promoters of genes encoding detoxification and antioxidant to protect mitochondria. We measured Nrf2 protein expression level in the cytosol and nucleus. The results showed that K141N-HSP8 overexpression significantly decreased Nrf2 protein level in the nuclear fraction (*p* < 0.05, Figure 5F), while NBP treatment increased Nrf2 protein level in K141N-HSPB8 group (*p* < 0.05, Figure 5F). Nrf2 protein level in the cytosolic fraction was not comparable among the groups. These results suggested that NBP could inhibit K141N-induced oxidative damage through activation of the Nrf2 pathway.

## Discussion

In the present study, we validated the impact of mutant HSPB8 on mitochondria dysfunction. Our study demonstrated that NBP has a protective effect on the neurotoxicity induced by HSPB8 K141N. Specifically, NBP has an antioxidant effect through activation of the Nrf2 pathway and could also modulate Bax/Bcl-2 proteins ratio. These findings suggest NBP may represent a potential therapy for patients with CMT2L.

The mitochondria play a pivotal role in contributing to defective energy metabolism, and their dysfunction is a hallmark of many neurodegenerative diseases (Johri and Beal, 2012; Lane et al., 2015). Six CMT2-associated genes have been associated with the mitochondria including: mitofusin 2 (MFN2), ganglioside-induced differentiation-associated protein 1 (GDAP1), kinesin family member 1B (KIF1B), low-molecular-weight neurofilament protein (NFL), heat shock protein 27 (HSP27), and dehydrogenase E1 and transketolase domain-containing 1 (DHTKD1) (Harding and Thomas, 1980; Xu W. Y. et al., 2012). Specifically, mutations in these genes impair mitochondrial transport and energy metabolism. Early passage fibroblast cells from two related dHMN2A patients with pathological HSPB8 mutations have highlighted the decreased mitochondrial membrane potential (Irobi et al., 2012), which suggests that mitochondrial dysfunction may play a critical role in the pathogenesis HSPB8-driven dHMN2A and CMT2L. However, the exact role of the mitochondria in the pathogenesis of CMT2L remains elusive. The aim of our study was primarily to investigate whether mitochondria dysfunction plays a role in the HSPB8 K141N driven CMT2L. We found mitochondrial aggregation in SH-SY5Y cells following overexpression of K141N HSPB8. We also observed aggregated mitochondria in sural nerves from CMT2L patient, yet such ultra structural findings were not found in diabetes patients, which is consistent with the TEM results from K141N-overexpressing cells. However, it is important to note that we cannot completely exclude the possibility that mitochondrial alternations in CMT2L patients are a consequence of age-related changes. Frankly, the use of a sample from one diabetic patient is insufficient as a control for specificity of the mechanisms studied herein, where neuropathy has multiple mechanisms and stages. We were unable to obtain sural nerves from normal controls and therefore searched previously published studies to confirm the specificity of the mitochondrial abnormalities observed. These changes were not reported in normal controls (Kalichman et al., 1998; Vallat et al., 2008). Further study from more CMT2L patients and healthy controls are needed to verify the results.

Ca^2+^ handling is controlled by mitochondrial shape and positioning. On the other hand, elevation of cytoplasmic and mitochondrial Ca^2+^ levels can disturb mitochondrial movements and modify their cellular distribution. Impaired Ca^2+^ buffering participates in the cell pathophysiology and pathogenesis of common neurodegenerative disorders (Lin and Beal, 2006; Szabadkai et al., 2006). The shape, motility and subcellular distribution of mitochondria are also dependent on the function of actin and microtubules (Foissner, 2004). Mutations in HSP27 exhibit enhanced interaction with actin and microtubules, leading to a reduction in microtubule dynamics (Almeida-Souza et al., 2011). HSPB8 is similar to HSP27 and has been shown to format hetero-oligomeric complexes with HSP27, which can interact with the mitochondrial outer membrane, and growing evidence has shown that K141N mutation in the HSPB8 protein stabilize its interaction with HSP27 (Benndorf et al., 2001; Sun et al., 2004). HSPB8 could also complex with the co-chaperone Bag3, which plays a role in actin dynamics (Fuchs et al., 2015). Whether HSPB8 K141N mutants could stabilize actin and microtubules still needs further investigation. Fission and fusion are essential processes that maintain mitochondrial distribution (Patrushev et al., 2015). Cells expressing MFN mutants have aggregated mitochondria. The mechanism of aggregation is hypothesized to be attributable to the formation of tethered intermediates which lead to imbalance of mitochondrial fission/fusion (Misko et al., 2012). The observation that K141N mutation resulted in an aggregation of the mitochondria suggests an involvement of K141N HSPB8 in regulating either the mitochondrial fusion, fission or trafficking. Whether mutant HSPB8 caused mitochondria aggregation is also result from abnormal mitochondria fission requires further studies.

Defects in mitochondrial fusion/fission have been shown to decrease mitochondrial movement and caused mitochondria aggregation (Patrushev et al., 2015; Bertholet et al., 2016) and abnormal fission or fusion could directly impacts mitochondrial function, for example resulting in excessive production of free radicals, altered mitochondrial enzymatic activities (Reddy, 2014). Generation of ROS is an inevitable outcome of the redox reaction. ROS can directly damage antioxidant enzymes such as superoxide dismutase (SOD), catalase (CAT) and reduce their activity, which will result in an increased accumulation of superoxide and hydroxyl radicals (Pigeolet et al., 1990; Zhang et al., 2014). When the production of free radicals increases abnormally or the defense mechanisms are damaged, accumulated ROS may cause serious cellular dysfunction. Indeed, we found that mutant HSPB8 increased ROS production, suppressed antioxidant enzyme levels (SOD and CAT) and the levels of the master transcription factor Nrf2 as compared with WT HSPB8. While treatment with NBP could inhibit K141N-induced oxidative damage by decreasing ROS production, in addition to radical scavenging, NBP could also enhance cellular resistance to intracellular oxidative stress by activation of the Nrf-2/ARE pathway as confirmed by the increased Nrf2 protein level in the nucleus and increased activities of SOD and CAT. As a novel agent for the treatment of stroke, NBP has been reported to have many neuronal protective effects in several diseases. However, no study has reported whether NBP could be beneficial as a treatment for HSPB8 K141N mutated CMT2L. In the present study, we demonstrated that NBP could ameliorate mitochondrial dysfunction by regulation of ROS generating and scavenging pathways, increased oxidative damage caused by K141N HSPB8 were reversed by treatment with NBP, similar to its effect on other diseases (Gao et al., 2010; Zhao et al., 2012).

By evaluating cell viability, we found that treatment with NBP for 24h suppressed K141N HSPB8-induced cell death in a dose-dependent manner. Another important finding was that NBP could inhibit HSPB8 K141N mutation-induced impact on neurite numbers in spinal motor neurons. The precise mechanism relating to how mutant HSPB8 induces neurite degeneration is unknown. Mitochondria, a coordinator for energy metabolism homeostasis contributes to many cellular functions of nervous system. Considering that the axon is sensitive to the mitochondrial energy supply, the decreased number of motor neurons may be attributable to mitochondria dysfunction and insufficient energy supply of the distal regions of peripheral axons. Indeed we found that HSPB8 K141N mutation caused mitochondrial aggregation and increased oxidative stress injury. Our results suggest that NBP could have a protection role on the mitochondria and alleviate HSPB8 K141N induced neuronal injury.

The mitochondrial pathway of apoptosis is classified into two different pathways, simply known as caspase-dependent and caspase-independent (Savitskaya and Onishchenko, 2015). Multiple studies have demonstrated that Bcl-2 family is involved in caspase-independent mitochondrial apoptosis pathways. The balance of pro-apoptotic proteins (e.g., Bax, Bad, Bid, and Bik) and anti-apoptotic proteins (e.g., Bcl-2, Bcl-w, and Bcl-xl) ultimately decides cell viability (Cory and Adams, 2002). Previous study from patient primary fibroblast showed that HSPB8-K141N mutation could elevate the vulnerability to pro-apoptotic stimuli in primary fibroblast culture (Irobi et al., 2012). To further evaluate the mechanisms by which HSPB8 K141N impacts mitochondrial dysfunction and the protective role of NBP, we examined the expression of mitochondrial apoptosis-related proteins (including Bcl-2 and Bax). We found that K141N mutants resulted in increased Bax protein expression, with concomitant down-regulation of Bcl-2. Pretreatment of NBP, however, enhanced cell viability by down-regulating the ratio of Bax/Bcl-2. These results suggest that the neurotoxicity of K141N HSPB8 may be attributable to the modulation of mitochondrial caspase-independent apoptosis pathway, while NBP exerted a protective effect.

In summary, our results demonstrate that mutant HSPB8 results in mitochondrial dysfunction, and that NBP has a protective effect on the neurotoxicity caused by this mutation. Our results will shed light on the pathogenesis of CMT2L and highlight the possibility of novel therapies. However, further *in vivo* studies are still needed to elucidate the effectiveness of NBP as a treatment for patients with CMT2L.

## Ethics statement

This study was carried out in accordance with the recommendations of Institutional Review Board from the Zhejiang University with written informed consent from all subjects. All subjects gave written informed consent in accordance with the Declaration of Helsinki. The protocol was approved by the Institutional Review Board from the Zhejiang University.

## Author contributions

XY and WL designed the project, organized the entire research. XY, ZC, KS, FX, and HW conceived the experiments. XY, ZC, and BL conducted the experiments. XY, JB, and ZC analyzed the results. XY wrote the manuscript. WL and HC revised the manuscript. All authors discussed the results and reviewed on the manuscript.

### Conflict of interest statement

The authors declare that the research was conducted in the absence of any commercial or financial relationships that could be construed as a potential conflict of interest.
